# Treatment of Acute Graft-versus-Host Disease in Liver Transplant Recipients

**DOI:** 10.1155/2021/8981429

**Published:** 2021-11-30

**Authors:** Edward Kim, Alina Adeel, Adel Bozorgzadeh, Shinya Amano, Curtis T. Barry, Jennifer S. Daly, Deepika Devuni, Zendee Elaba, Laura Houk, Paulo N. Martins, Babak Movahedi, Muthalagu Ramanathan, Nicole M. Theodoropoulos

**Affiliations:** ^1^Department of Surgery, UMass Memorial Medical Center, Worcester, MA, USA; ^2^Department of Medicine, Division of Infectious Diseases & Immunology, UMass Memorial Medical Center, Worcester, MA, USA; ^3^Department of Surgery, Division of Organ Transplantation, UMass Memorial Medical Center, Worcester, MA, USA; ^4^Department of Pathology, UMass Memorial Medical Center, Worcester, MA, USA; ^5^Department of Medicine, Division of Gastroenterology, UMass Memorial Medical Center, Worcester, MA, USA; ^6^Department of Dermatology, UMass Memorial Medical Center, Worcester, MA, USA; ^7^Department of Medicine, Division of Hematology and Oncology, UMass Memorial Medical Center, Worcester, MA, USA

## Abstract

Acute graft-versus-host disease (aGvHD) is a rare complication of liver transplantation associated with high morbidity and mortality. Death typically occurs due to complications related to severe infection, shock, and multiorgan failure. The clinical presentation involves dysfunction of multiple organ systems with overlapping symptoms that often results in a diagnostic delay. As there are a limited number of cases reported in the literature, there are no clear guidelines for treatment. Many different therapeutic measures have been utilized that target various immune system pathways, but steroids remain the first line of therapy. We report on two patients who developed aGvHD after liver transplantation who were treated with ruxolitinib, a novel Janus kinase 1/2 (JAK) inhibitor that has been shown to improve outcomes in steroid refractory cases of aGvHD after allogenic hematopoietic stem cell transplantation. We reviewed the literature to discuss various therapeutic options currently available for aGvHD after liver transplantation.

## 1. Introduction

Acute graft-versus-host disease (aGvHD) is a well-known complication of allogeneic hematopoietic stem cell transplantation (HSCT) [1]. It is less commonly observed after solid organ transplantation (SOT) and occurs most frequently after intestinal or multivisceral transplantation [2]. Acute GvHD after liver transplantation is a rare condition with an incidence of up to 2% and a very high mortality due to delay in diagnosis and a lack of standard therapeutic regimen [3]. The mechanism for aGvHD after SOT is not well defined. It is postulated that the immunocompetent T-cells from the graft may be the culprit, ultimately undergoing activation and ensuing tissue damage in the host. We describe two cases of aGvHD after liver transplantation and review the use of ruxolitinib for aGvHD after SOT.

## 2. Case Description

### 2.1. Case 1

**Figure 1:**
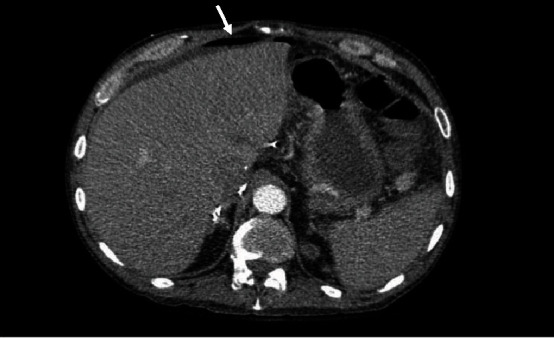
Axial abdominal CT angiogram showing free air in peritoneal cavity (white arrow) secondary to perforated duodenum.

The patient was a 60-year-old man with history of cirrhosis due to alcohol associated liver disease (ALD) and hepatocellular carcinoma (HCC), who underwent deceased donor liver transplantation (DDLT) in 2019 with methylprednisolone induction. He was ABO blood group O, seronegative for cytomegalovirus (CMV) with a Model for End-Stage Liver Disease (MELD) score of 25 at the time of transplantation. The donor was a 66-year-old man with ABO blood group O, also seronegative for CMV. One month after transplantation, the patient presented to the hospital with acute onset of severe abdominal pain, nonbloody diarrhea, and decreased urinary output for the past 3 days. His immunosuppression regimen on admission consisted of mycophenolate mofetil 500 mg twice daily, tacrolimus with trough level of 9.1 mcg/L, and prednisone 20 mg daily. He was in shock on presentation requiring vasopressors, and empiric intravenous (IV) piperacillin-tazobactam, IV vancomycin, IV metronidazole, and oral vancomycin were initiated. The liver enzymes were normal at the time of presentation except for mildly elevated alkaline phosphatase at 123 U/L. The metabolic panel was significant for hyperkalemia, hyponatremia, acute kidney injury, and severe metabolic acidosis. An abdominal CT scan showed pneumoperitoneum and suspicion for duodenal perforation (Figure 1); he was emergently taken to the operating room for repair of a perforated ulcer in the first part of duodenum.

**Figure 2:**
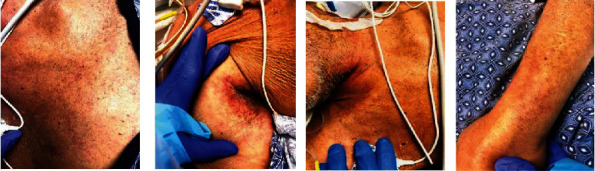
Petechial rash on trunk, axilla, neck, and arm.

**Figure 3:**
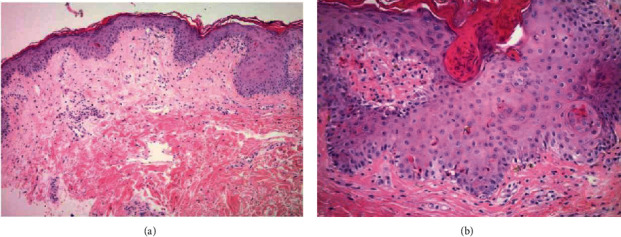
(a) Punch biopsy showing paucicellular lymphocytic infiltrate within the dermis (hematoxylin-eosin, original magnification ×100). (b) Dyskeratotic cells in the epidermis (hematoxylin-eosin, original magnification ×200).

Postoperatively, he remained in shock requiring multiple vasopressors and stress dose steroids. The antibiotics were changed to IV meropenem, IV tobramycin, and IV micafungin. The following day, he developed neutropenia with an absolute neutrophil count (ANC) of <100 cells/*μ*L, and granulocyte colony stimulating factor (G-CSF) was started. Mixed chimerism studies were sent on hospital day 3 as GvHD was suspected. A new petechial rash was noted on his legs and arms on hospital day 7 (Figure 2). The myeloid and lymphoid chimerism studies came back the same day with >21% donor DNA and 20% donor cells consistent with GvHD. Based on these results, the immune suppression was augmented with tacrolimus, mycophenolate, and methylprednisolone 2 mg/kg/day. The skin biopsy was significant for interface dermatitis with dyskeratotic keratinocytes consistent with the diagnosis of aGvHD (Figure 3).

Ruxolitinib was initiated given the lack of clinical response to steroids after 4 days of therapy. Unfortunately, he had persistent neutropenia, worsening skin rash, gastrointestinal bleeding, and persistent shock despite multiple vasopressors. Due to multiorgan failure despite maximal medical support, the goals of care were changed per patient's and family's wishes, and he died on day 16 of admission, 45 days after transplant.

### 2.2. Case 2

**Figure 4:**
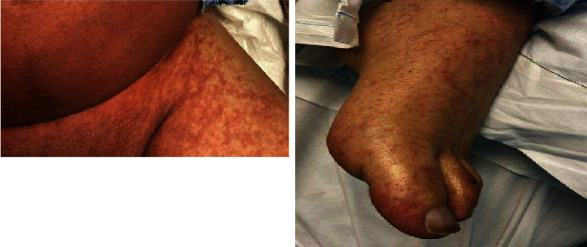
Maculopapular rash on trunk and legs.

The patient was a 67-year-old man with a history of cirrhosis due to ALD and HCC who underwent DDLT in 2020 with methylprednisolone induction. He was ABO blood group O, seronegative for CMV with a MELD score of 33 at the time of transplantation. The donor was a 23-year-old man with ABO blood group A, also seronegative for CMV. He returned to the hospital 15 days posttransplant with fever and diarrhea. His immunosuppression regimen on admission consisted of mycophenolate mofetil 500 mg twice daily, tacrolimus with trough level of 8.6 mcg/L, and prednisone 20 mg daily. The initial work-up was notable for *Clostridioides difficle* toxin detectable by stool PCR; oral vancomycin was started. Liver enzymes were normal at the time of presentation. Fevers continued with development of abdominal distention on day 3 of admission. An abdominal CT scan revealed generalized mesenteric and soft tissue edema and small volume ascites. At the same time, he became neutropenic with ANC of 0 cells <*μ*L. Later that day, the patient developed refractory shock requiring vasopressors and acute hypoxic respiratory failure requiring endotracheal intubation and mechanical ventilation. Antimicrobial coverage was broadened to include IV cefepime and IV metronidazole. The following day, he developed a diffuse maculopapular rash (Figure 4).

**Figure 5:**
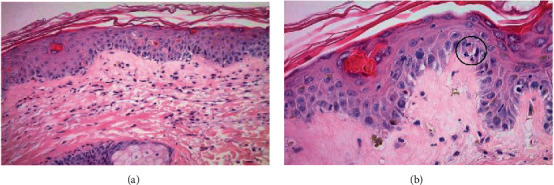
(a) Dyskeratotic cells in the epidermis (hematoxylin-eosin, original magnification ×200). (b) Lymphocytes in close apposition to apoptotic keratinocytes (satellite cell necrosis), (hematoxylin-eosin, original magnification ×400).

A skin biopsy and peripheral blood chimerism studies were done on hospital day 4 due to concern for aGvHD. Renal replacement therapy was initiated for acute renal failure. On hospital day 7, the chimerism studies resulted with 92% donor cells, and skin biopsy was significant for vacuolar interface dermatitis with dyskeratotic keratinocytes (Figure 5) consistent with aGVHD.

**Figure 6:**
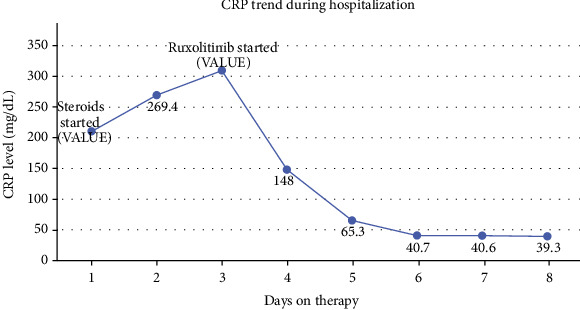
C-reactive protein trend during hospitalization for case 2.

Immunosuppression was expanded to add high dose steroids with methylprednisolone 1 mg/kg every 12 hours along with increase in tacrolimus dose. On hospital day 7, GvHD-related laboratory markers were noted to be elevated (Table 1). Ruxolitnib was initiated due to no clinical response to steroids after two days. On ruxolitinib, the rash and ANC improved with a decline in C-reactive protein (CRP) level (Figure 6).

On day 14 of admission, he developed septic shock secondary to vancomycin resistance *Enterococcus faecium* bacteremia; IV daptomycin was started. Despite maximal medical management, he continued to deteriorate with multiorgan failure, and goals of care were changed based on the patient's and family's wishes. The patient died 29 days after transplant.

## 3. Discussion

These case reports illustrate the challenges in diagnosis and treatment of aGvHD and fatal outcomes in two patients who developed aGvHD after liver transplantation despite treatment with steroids and ruxolitinib. There is a paucity of aGvHD cases after SOT that are treated with ruxolitinib with a recent case series of only three patients with aGvHD after SOT demonstrating good response to ruxolitinib in two of the patients [4]. No other cases of ruxolitinib use for aGvHD after SOT have been reported in the literature to date. Both of our patients were treated with ruxolitinib with one showing some improvement in clinical parameters but had no change in mortality related to infection.

Acute GvHD after SOT is relatively uncommon and carries a mortality of up to 80% after liver transplantation [3]. It is thought to be a T-cell mediated response which occurs when immunocompetent donor-derived T-cells undergo activation, IL-2-dependent clonal expansion, and differentiation into effector T-cells that get activated and cause apoptosis of host target cells [5]. The native immune system remains in play while the donor-derived T-cells cause multisystem tissue damage in the host, with most susceptible tissues being skin, liver, gastrointestinal (GI) tract, and bone marrow. Importantly, the transplanted graft is not affected.

Acute GvHD typically presents within 3 to 5 weeks after liver transplantation with clinical features including skin rashes or mucosal lesions, fever, diarrhea or other GI symptoms, and pancytopenia [3]. Although fever and diarrhea can be a presentation of aGvHD, an infection can also present with similar symptoms and could have instigated aGVHD. In both of our patients, septic presentation preceded pancytopenia. Therefore, we believe that both sepsis and GvHD contributed to the pancytopenia. Duodenal perforation as initial presentation of aGvHD, as observed in our patient, is exceedingly rare and previously only reported in the pediatric population [6]. The diagnosis of GvHD typically involves peripheral blood macrochimerism studies, skin biopsy, and GI biopsy in conjunction with the clinical presentation [3].

High dose steroids remain the first line of therapy for aGvHD, but reports do not show improvement in overall mortality [7]. Although the term “steroid refractory GvHD” is defined for HSCT patients, there is a lack of a consistent standard duration of steroid use in aGvHD for SOT patients. Other potential therapies include IL-2 and TNF-*α* blockers, such as daclizumab, infliximab, and etanercept which have been utilized in conjunction with steroids as well as used as monotherapy, but overall outcomes remain poor [8, 9]. Alefacept, which prevents the activation of CD4 and CD8 T-lymphocytes and antithymocyte globulin, eliminates the activated effector T-cells, when used in combination with high dose steroids in patients who develop pancytopenia that have been shown to result in immediate rebound of bone marrow function, but with increased risk of infection [10, 11]. Other options include extracorporeal photopheresis, which modulates the immune system and was found in one report to decrease peripheral blood chimerism but had no effect on mortality [12]. Another therapeutic modality includes significant reduction in immunosuppression, allowing the native immune system to reject donor T-lymphocytes. However, reducing or withdrawing immunosuppression may confer risk for graft rejection and possibly worsening GvHD as donor T-cells are liberated from inhibition induced by the immunosuppressive agents [5].

Activated Janus kinases (JAK) are required for effector T-cell responses in GvHD after HSCT. Ruxolitinib is an oral selective JAK 1/2 inhibitor with equipotent activity towards JAK1 and JAK2. Inhibition of JAK1/2 signaling resulted in the reduced proliferation of effector T-cells with a clinical response rate of 81.5% in patients with steroid refractory GvHD in allogeneic HSCT [13, 14]. Ruxolitinib was demonstrated to have potent activity in steroid refractory aGvHD after allogeneic HSCT compared to other agents [15], but only three cases of use in SOT patients have been reported thus far [4].

Successful treatment of aGvHD after liver transplantation remains challenging with limited data and no consensus on the most appropriate therapy [16]. Novel agents are on the horizon, but more robust data are needed to establish drugs like ruxolitinib as the preferred agent for aGvHD after SOT. The major pitfall of increasing immunosuppression for treatment of GvHD is the increased risk of life-threatening infections. T-cell depletion in selected cases of HSCT has been shown to reduce the risk of acute and chronic GvHD [17], but this modality has not been reported in liver transplantation. Given the poor outcomes of aGVHD and limited therapeutic options, research in that direction should be explored. In addition to immune targeted therapy, intensive prophylactic and empiric antimicrobial therapy remains crucial to prevent and minimize mortality and morbidity related to infectious complications. It is critical to identify patients with possible GvHD as soon as possible by recognizing the signs of preserved graft function, bone marrow suppression or pancytopenia, GI symptoms of diarrhea, and skin toxicity mainly manifested as skin rash and to start treatment as early as possible.

## Figures and Tables

**Table 1:** GVHD-specific markers prior to initiation of ruxolitinib.

Test name	Result	Normal range
Elafin	>80 ng/mL	4.9–23.8 ng/mL
ST 1: soluble TNF-receptor 1	>9.8 ng/mL	<2.5 ng/mL
Reg 3 alpha: regenerating islet-derived 3-alpha	195.5 ng/mL	<89.0 ng/mL
ST 2: soluble TNF-receptor 2	>100.0 ng/mL	<30 ng/mL

## Data Availability

No data were used to support this study.

## Abbreviations

ALD: Alcohol associated liver disease
aGvHD: Acute graft-versus-host disease
ANC: Absolute neutrophil count
CMV: Cytomegalovirus
CRP: C-reactive protein
DDLT: Deceased donor liver transplantation
GI: Gastrointestinal
G-CSF: Granulocyte colony stimulating factor
GvHD: Graft-versus-host disease
HCC: Hepatocellular carcinoma
HSCT: Hematopoietic stem cell transplantation
IV: Intravenous
JAK 1/2: Janus kinase
MELD: Model for end-stage liver disease
SOT: Solid organ transplant.
